# Association between coronary artery calcium scores and retinal ischemic perivascular lesions

**DOI:** 10.1186/s40942-026-00798-2

**Published:** 2026-02-21

**Authors:** Jay Bharatsingh Bisen, Hayden Sikora, Michael Drakopoulos, John Bryan, Maxwell Shramuk, Adin-Cristian Andrei, Richard L. Weinberg, Rukhsana G. Mirza

**Affiliations:** 1 Department of Ophthalmology, Northwestern University Feinberg School of Medicine, 645 N Michigan Ave Suite 440, Chicago, IL 60611 USA; 2 Biostatistics Collaboration Center, Northwestern University Feinberg School of Medicine, Chicago, IL USA; 3 Department of Medicine, Division of Cardiology, Northwestern University Feinberg School of Medicine, Chicago, IL USA

**Keywords:** Retinal ischemic perivascular lesions, Optical coherence tomography, Coronary artery calcium score, Oculomics

## Abstract

**Background:**

Coronary artery calcium (CAC) scores measure atherosclerosis and are associated with increased cardiovascular disease (CVD) risk. Retinal ischemic perivascular lesions (RIPLs) represent focal retinal infarcts and have been linked to systemic CVD. Given their association with retinal microcirculation dysfunction, RIPLs might serve as early indicators of systemic vascular dysfunction. This study aims to assess the relationship between CAC scores and RIPLs.

**Methods:**

Retrospective, single-institution, cross-sectional study. Patients consenting for research who had both high-quality, bilateral, macular optical coherence tomography (OCT) imaging and a CAC score of either 0 (low risk) or ≥ 300 (high risk) within one year after their macular OCT imaging. RIPLs were identified as regions of focal inner nuclear layer (INL) thinning, outer plexiform layer (OPL) inward deviation, and outer nuclear layer (ONL) expansion on OCT B-scans in the absence of underlying or overlying retinal pathology. The difference in mean total RIPLs per patient between the high- and low-risk groups was assessed. The percentages of individuals in each group with high RIPL count (≥ 2) were compared as well. The effects of potential confounders (known cardiovascular risk factors) on the relationship between RIPL counts and CAC-score-based risk group were examined with multivariate analysis.

**Results:**

90 patients (low-risk: 66; high-risk: 24) were included. High-risk patients were older (mean ± std of 72.1y ± 7.7 vs. 62.1y ± 10.5; *p* < 0.001), more likely male (46% vs. 17%; *p* = 0.004), and were more likely to have hypertension (79% vs. 30%; *p* < 0.001), type 2 diabetes mellitus (38% vs. 9%; *p* = 0.003), smoking history (62% vs. 22%; *p* = 0.021), atrial fibrillation (17% vs. 0%; *p* = 0.004), and lower total cholesterol (155.2 mg/dL ± 36.3 vs. 201.0 mg/dL ± 41.2; *p* < 0.001). Increased raw RIPL counts (5.29 ± 7.54 vs. 2.36 ± 3.52; *p* = 0.008) and increased probability of elevated RIPL presence (75% with ≥ 2 RIPLs vs. 48%; *p* = 0.025) were observed in high-risk patients.

**Conclusions:**

CAC score ≥ 300 is associated with an increased number of RIPLs and an increased probability of elevated RIPL presence (≥ 2 RIPLs), potentially mediated by known CVD risk factors. RIPLs might identify those with elevated CVD risk, and RIPLs might suggest presence of established CVD risk factors.

## Introduction

Cardiovascular disease (CVD) remains a leading cause of morbidity and mortality worldwide. CVD has increasing prevalence [1, 2], underscoring the importance of CVD screening methods. The Pooled Cohort Equation to estimate Atherosclerotic Cardiovascular Disease (ASCVD) and the American Heart Association (AHA) Predicting Risk of CVD Events (PREVENT) risk calculators and noninvasive-imaging-derived metrics, such as coronary artery calcium (CAC) scoring, have emerged as valuable tools in CVD screening and risk stratification, offering insights into a patient’s atherosclerotic burden and overall cardiovascular risk [3–5]. However, while these tools are predictive of adverse outcomes, they may lack sensitivity for early microvascular changes that precede overt coronary artery disease and its complications [6, 7].

Given this information, there is a pressing need for the development of novel and sensitive noninvasive tools to offer robust insights into systemic microvascular function. Optical coherence tomography (OCT) and OCT-angiography (OCTA) are powerful, noninvasive imaging modalities for visualizing the retinal microvasculature [8, 9]. OCT enables detailed cross-sectional imaging of the retina, capturing its individual layers with precision, while OCTA allows visualization of the vascular networks within each retinal layer, including the superficial (SCP), intermediate (ICP), and deep (DCP) capillary plexuses. OCT and OCTA imaging can visualize structural manifestations of ischemic disease at various stages of the ischemic cascade [10], including cotton wool spots, paracentral acute middle maculopathy (PAMM), and retinal ischemic perivascular lesions (RIPLs) [11].

RIPLs, an emerging retinal imaging biomarker, represent focal infarcts of the middle retina, thought to be secondary to middle retina hypoperfusion or ischemia. RIPLs can be identified on OCT imaging as areas of outer nuclear layer expansion, outer plexiform layer inward displacement, and inner nuclear layer thinning [11]. Originally identified as a permanent legacy of diminutive (PAMM) [12–15], RIPLs are often present in patients without known systemic ischemic disease and without symptoms [15]. Given their utility as a subclinical biomarker of retinal infarction, RIPLs have gained particular interest for their association with cardiovascular diseases that may affect the retinal microvasculature [11, 16]. Various studies have associated RIPLs with comorbid hypertension [12], chronic cardiovascular disease [16], stroke [16–18], carotid artery stenosis [19, 20], myocardial infarction [21], and atrial fibrillation [22]. RIPLs may even more generally reflect the risk of cardiovascular events, as patients with a higher 10-year ASCVD risk score were found to correlate with a higher number of RIPLs [16].

Although RIPLs have been associated with CVD, their role in clinical practice, whether as a predictor of CVD risk or as a marker of subclinical disease progression, remains to be fully characterized. CAC scores are well-established markers associated with increased ASCVD risk, providing a direct measure of atherosclerotic burden within the coronary arteries [23]. CAC scores are derived from computed tomography (CT) scans of the heart and reflect calcifications of the coronary arteries. The specific numerical metric is a weighted average of the volume of the coronary artery calcifications noted on the CT scan and the radiodensity of those calcifications. A score of zero is only assigned when no calcification is identified. CAC score shows equivalence with total coronary atherosclerotic load and is associated with increased ASCVD risk scores [24, 25]. Various studies have demonstrated that a higher CAC score is associated with a higher risk of major cardiovascular events, including nonfatal myocardial infarction, cardiac mortality, and all-cause mortality. Studies also demonstrate that individuals with CAC > 300, even without prior ASCVD, experience similar rates of major adverse cardiovascular events, myocardial infarction, and mortality compared to those with known ASCVD, with around 20% experiencing events over a median four-year follow-up [25]. Notably, CAC scores ≥ 300 are significantly associated with 10-year ASCVD event rates as high as 25.6% [25–27].

Given their established associations with CVD and their association with retinal microvascular dysfunction, RIPLs might serve as early indicators of systemic vascular dysfunction, similar to CAC score. This study aims to assess the possible relationship between CAC scores and RIPLs, including accounting for baseline differences in CVD risk factors with multivariate analysis.

## Methods

This study adhered to the tenets of the Declaration of Helsinki and to HIPAA regulations, and Institutional Review Board approval was obtained from Northwestern University in Chicago, Illinois. Patients with informed consent for research were included. Patient data was de-identified to ensure privacy and confidentiality.

**Fig. 1:**
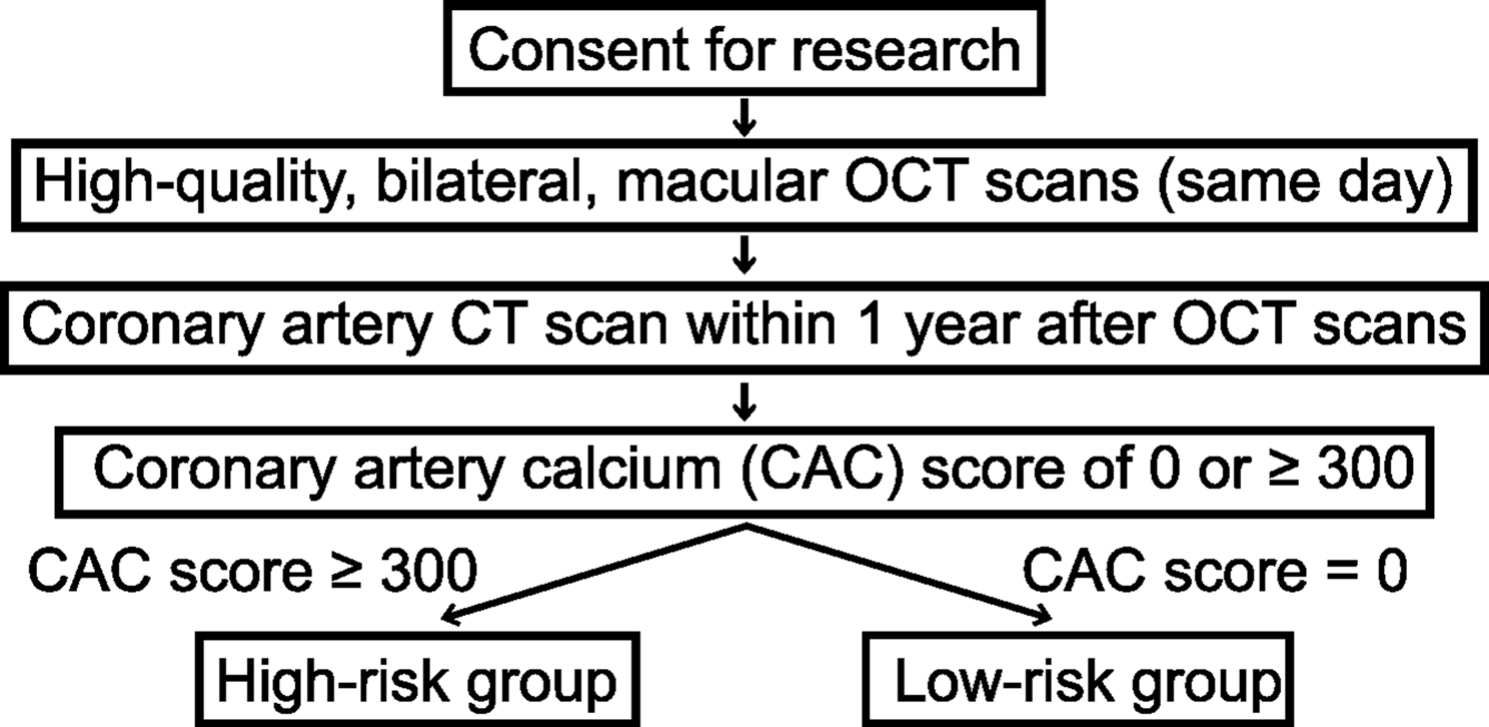
Flow diagram of patient selection. Patients consenting for research who had both high-quality (not low resolution or with motion or shadow artifact), bilateral, macular OCT imaging on a single day and a coronary artery CT score of either 0 (low risk) or ≥ 300 (high risk) within one year after their macular OCT imaging were identified as the high-risk and low-risk groups

This retrospective, single-institution cross-sectional study included all patients who underwent both bilateral retinal OCT imaging and a coronary artery CT scan within one year after retinal OCT imaging. Retinal OCT imaging consisted of high-quality 6 mm by 6 mm fovea-centered macular OCT volume scans with 61 OCT B-scans each (Spectralis, EssilorLuxottica, Paris, France) taken on the same day. Patients with poor-quality OCT images, defined as those with low resolution, motion artifact, or shadow artifact, were excluded. CAC scoring was conducted using the Agatston scoring method, with scores extracted directly from electronic health records. Patients were categorized into two groups based on CAC scores: those with CAC = 0 (low risk) and those with CAC ≥ 300 (high risk). Intermediate CAC scores (1–299) were excluded to enable a direct comparison between high and low-risk groups. A flow diagram for patient classification is included in Fig. 1.

**Fig. 2:**
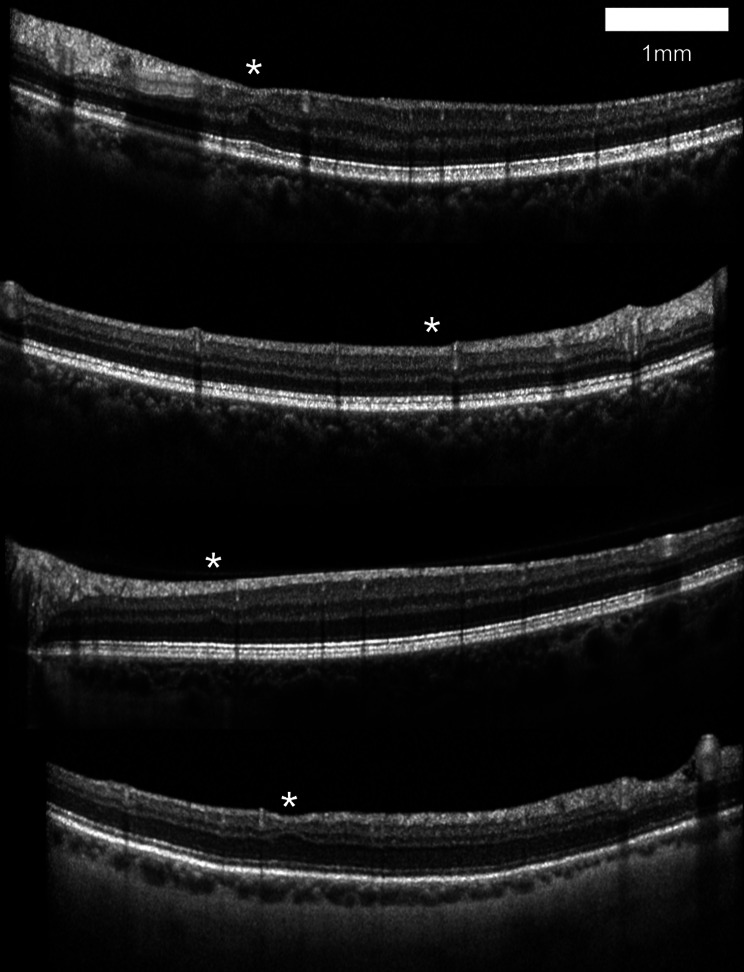
Four 6 mm-wide spectral domain optical coherence tomography B-scans with examples of retinal ischemic perivascular lesions (RIPLs) identified in the current study. RIPLs are marked by an asterisk (“*”) placed above the center of each RIPL. One-millimeter scale bar for width as shown

All OCT B-scans from the included OCT images were graded for RIPLs independently by two experienced graders (JB, HS), with 72% interrater agreement and discrepancies resolved by a third grader (RM), a medical retina specialist, a similar method employed in previous studies [28–30]. RIPL counts were the summed RIPL counts between both eyes. All graders had prior experience with RIPL grading on SD-OCT. RIPLs were defined as region of focal inner nuclear layer (INL) thinning, outer plexiform layer (OPL) inward deviation, and outer nuclear layer (ONL) expansion on OCT B-scans in the absence of underlying or overlying retinal pathology, consistent with prior work [11–22]. RIPLs that were identified on multiple consecutive B-scans were counted only once. Example images of identified RIPLs are presented in Fig. 2.

Clinical and demographic data, including age, sex, hypertension (HTN), type 2 diabetes mellitus (T2DM), smoking status, stroke history, chronic kidney disease (CKD), peripheral arterial disease (PAD), atrial fibrillation (AF), carotid artery stenosis (CAS), hypercholesterolemia, cholesterol levels (closest blood draw to retinal OCT imaging), anti-hypertensive use [Chlorthalidone, Hydrochlorothiazide, Lisinopril, Enalapril, Ramipril, Benazepril, Losartan, Valsartan, Azilsartan, Amlodipine, Nifedipine, Diltiazem, Verapamil, Furosemide, Torsemide, Spironolactone, Eplerenone, Metoprolol succinate, Atenolol, and Carvedilol], anti-coagulant use [Warfarin, Dabigatran, Apixaban, Rivaroxaban, Edoxaban, Betrixaban, Enoxaparin, Dalteparin, Heparin, Fondaparinux, and Argatroban], and lipid lowering medication use [Atorvastatin, Rosuvastatin, Simvastatin, Pravastatin, Ezetimibe, Evolocumab, Fenofibrate, Gemfibrozil, and Niacin], were extracted from the electronic medical records (EMR). Comorbidities were determined based on medical history and diagnoses documented within the EMR.

### Statistical analysis

Clinical and demographic characteristics and RIPL counts were compared between the high- and low-risk groups. Categorical variables, such as the percentage of patients with HTN, T2DM, stroke history, CKD, PAD, AF, CAS, and hypercholesterolemia, were compared using Chi-squared or Fisher’s exact testing between cohorts. Continuous variables such as cholesterol levels were compared using Wilcox rank sum testing.

RIPL counts were compared using Pearson’s Chi-squared test for categorical comparisons of raw RIPL counts between risk groups and the Wilcoxon rank sum test for assessing differences in the distribution of RIPL scores as continuous variables per individual. Previous studies have identified a significant increase in the odds of patients with greater than 1 RIPL having cardiovascular disease [16]. Therefore, total RIPL counts were also stratified into categorial groups [Low RIPL Count: 0–1; High RIPL Count: 2+] and analyzed using Fisher’s exact testing for categorical comparisons. Univariate and Multivariate analyses were performed using logistic regression models to examine the relationship between total RIPL scores (2 categories: 0–1 vs. 2+) and cardiovascular risk. Multivariate analyses adjusted for potential confounders, including age, T2DM, hypertension, and smoking status. LASSO regression techniques were applied to determine the best-fitting model. P-values < 0.05 were considered statistically significant.

## Results

A total of 90 patients were included in the study, with 66 classified as low risk (CAC = 0) and 24 as high risk (CAC ≥ 300). High-risk patients were significantly older (mean age: 72.1 vs. 62.1 years, *p* < 0.001) and had a higher proportion of males (46% vs. 17%, *p* = 0.004) (Table 1). They also had higher odds of hypertension (79% vs. 30%, *p* < 0.001), T2DM (38% vs. 9.1%, *p* = 0.003), and atrial fibrillation (17% vs. 0%, *p* = 0.004), as well as a history of smoking (current or former) compared to low-risk patients (62% vs. 32%, *p* = 0.021) (Table 1). High-risk patients interestingly had significantly lower total cholesterol levels than low-risk patients (mean: 155.2 vs. 201.0 mg/dL, *p* < 0.001) (Table 1). Lastly, high-risk patients had elevated anti-hypertensive (88% vs. 32%, *p* < 0.001) and lipid lowering medication (75% vs. 29%, *p* < 0.001) medication use compared to low-risk patients (Table 1).

**Table 1:** Baseline characteristics of the overall cohort, with a comparison of demographic characteristics between the low-risk group (CAC = 0) and the high-risk group (CAC ≥ 300)

Characteristic		Overall, *N* = 90 ^1^	Low Risk, *N* = 66 ^1^		High Risk, *N* = 24 ^1^	*P* value
**Age** ^2^		64.8 ± 10.8	62.1 ± 10.5		72.1 ± 7.7	< 0.001
**Gender** ^3^						0.004
Male		22 (24%)	11 (17%)		11 (46%)	
Female		68 (76%)	55 (83%)		13 (54%)	
**Race** ^3^						0.4
Non-African American		82 (91%)	61 (92%)		21 (88%)	
African American		8 (9%)	5 (8%)		3 (13%)	
**Hypertension** ^3^						< 0.001
No		51 (57%)	46 (70%)		5 (21%)	
Yes		39 (43%)	20 (30%)		19 (79%)	
**Blood Pressure Classification** ^4^						0.7
Normal		31 (34%)	25 (38%)		6 (25%)	
Elevated		11 (12%)	8 (12%)		3 (13%)	
Stage 1 HTN		27 (30%)	19 (29%)		8 (33%)	
Stage 2 HTN		21 (23%)	14 (21%)		7 (29%)	
**Type 2 Diabetes Mellitus** ^3^						0.003
No		75 (83%)	60 (91%)		15 (62%)	
Yes		15 (17%)	6 (9%)		9 (38%)	
**Smoking Status** ^4^						0.021
Never		54 (60%)	45 (68%)		9 (38%)	
Former		33 (37%)	19 (19%)		14 (58%)	
Current		3 (3%)	2 (3%)		1 (4%)	
**Stroke History** ^3^						0.9
No		89 (99%)	65 (98%)		24 (100%)	
Yes		1 (1%)	1 (2%)		0 (0%)	
**Chronic Kidney Disease** ^3^						0.056
No		86 (96%)	65 (98%)		21 88%)	
Yes		4 (4%)	1 (2%)		3 (13%)	
**Peripheral Artery Disease** ^3^						0.069
No		88 (98%)	66 (100%)		22 (92%)	
Yes		2 (2%)	0 (0%)		2 (8%)	
**Atrial Fibrillation** ^3^						0.004
No		86 (96%)	66 (100%)		29 (83%)	
Yes		4 (4%)	0 (0%)		4 (17%)	
**Hypercholesterolemia** ^3^						0.5
No		50 (56%)	38 (58%)		12 (50%)	
Yes		40 (44%)	28 (28%)		12 (50%)	
**Carotid Artery Stenosis** ^3^						0.9
No		86 (96%)	63 (95%)		23 (96%)	
Yes		4 (4%)	3 (5%)		1 (4%)	
**History of CVD** ^3^						< 0.001
No		60 (67%)	55 (83%)		5 (21%)	
Yes		30 (33%)	11 (17%)		19 (79%)	
**Total Cholesterol** ^2^		188.6 ± 44.7	201.0 ± 41.2		155.2 ± 36.3	< 0.001
**Anti-Hypertensive Used** ^3^						< 0.001
No		48 (53%)	45 (68%)		3 (12%)	
Yes		42 (47%)	21 (32%)		21 (88%)	
**Lipid Lowering Medication Used** ^3^						< 0.001
No		53 (59%)	47 (71%)		6 (25%)	
Yes		37 (41%)	19 (29%)		18 (75%)	
**Anti-Coagulant Used** ^3^						0.10
No		77 (86%)	59 (89%)		18 (75%)	
Yes		13 (14%)	7 (11%)		6 (25%)	

^1^ Mean ± (SD); n (%)^2^ Wilcox rank sum test^3^ Fisher’s exact test^4^ Pearson’s Chi-squared test

Wilcox rank sum testing identified statistically significant differences between the mean RIPL counts between high and low-risk groups (*p* = 0.008), however Pearsons Chi-squared testing showed no significant differences between the RIPL distribution between the two groups (*p* = 0.086). Using a two-category classification (0–1 vs. ≥2 RIPLs), the high-risk group showed a significantly higher proportion of patients with elevated RIPL scores (≥ 2) compared to the low-risk group (63% vs. 33%, *p* = 0.025). When using a three-category classification (0–1, 2–4, ≥ 5 RIPLs), a higher proportion of high-risk patients were observed in the ≥ 5 RIPL category compared to the low-risk group (33% vs. 11%, *p* = 0.018) (Table 2).

**Table 2:** Comparison of retinal ischemic perivascular lesions between the high-risk group (CAC ≥ 300) and the low-risk group (CAC = 0)

Characteristic		Overall, *N* = 90^1^	Low Risk, *N* = 66^1^		High Risk, *N* = 24^1^	*P* value
**RIPL Score** ^2^		3.14 ± 5.04	2.36 ± 3.52		5.29 ± 7.54	0.008
**RIPL Distribution** ^3^		3.14 ± 5.04	2.36 ± 3.52		5.29 ± 7.54	0.086
**Categorized total RIPL score (2 categories)** ^4^						0.025
0–1		40 (44%)	34 (52%)		6 (25%)	
2+		50 (56%)	32 (48%)		18 (75%)	
**Categorized total RIPL score (3 categories)** ^3^						0.018
0–1		40 (44%)	34 (52%)		6 (25%)	
2–4		35 (39%)	25 (38%)		10 (42%)	
5+		15 (17%)	7 (11%)		8 (33%)	

^1^ Mean ± (SD); Median (IQR); n (%)^2^ Wilcox rank sum test^3^ Pearson’s Chi-squared test^4^ Fisher’s exact test

Univariate logistic regression demonstrated a statistically significant association between high CAC scores and elevated RIPL scores (≥ 2) with an odds ratio (OR) of 3.19 (95% CI: 1.17–9.72, *p* = 0.029) (Table 3). However, in multivariate analyses adjusting for age, biological sex, T2DM, hypertension, smoking, anti-hypertensive medication use, and lipid lowering medication use, no independent relationship was observed between CAC scores and total RIPL counts (OR: 2.25, 95% CI: 0.56–9.37, *p* = 0.30) (Table 4). Excluding hypertension from the model yielded similar findings, with no statistically significant association between CAC scores and RIPL counts (OR: 2.04, 95% CI: 0.52–8.30, *p* = 0.30).

**Table 3:** Univariate logistic regression models assessing the relationship between total RIPL score between low-risk (CAC = 0) and the high-risk groups (CAC ≥ 300)

Population		Odds Ratio		95% Confidence Interval	*P* value
Low Risk		-		-	
High Risk		3.19		1.17–9.72	0.029

**Table 4:** Multivariate logistic regression models assessing the relationship between total RIPL score between low-risk (CAC = 0) and the high-risk groups (CAC ≥ 300)

Characteristic		Odds Ratio		95% Confidence Interval	*P* value
Population					
Low Risk		-		-	
High Risk		2.25		0.56–9.37	0.30
**Age**		1.01		0.97–1.06	0.60
**Biological Sex**					
Male		-		-	
Female		1.14		0.36–3.92	0.80
**Type-2 Diabetes Mellitus**					
No		-		-	
Yes		2.66		0.69–11.20	0.20
**Hypertension**					
No		-		-	
Yes		0.48		0.08–2.32	0.40
**Smoking**					
Never		-		-	
Former		0.49		0.16–1.41	0.20
Current		1.78		0.12–46.60	0.70
**Anti-Hypertensive Medication Use**					
No		-		-	
Yes		2.41		0.48–14.70	0.30
**Lipid Lowering Medication Use**					
No		-		-	
Yes		1.40		0.46–4.18	0.50

LASSO regression identified age and biological sex as the only variables retained in the final model alongside CAC risk categories. The adjusted OR for elevated RIPL scores in the high-risk group remained non-significant (OR: 3.27, 95% CI: 0.59–18.64, *p* = 0.14). Age and biological sex were also not significantly associated with RIPL counts in the final model (OR: 1.00, 95% CI: 0.94–1.07, *p* = 0.93; OR: 1.18, 95% CI: 0.21–4.84, *p* = 0.85 respectively) (Table 5).

**Table 5:** Results of the optimal model selected using LASSO regression

Characteristic		Odds Ratio		95% Confidence Interval	*P* value
**Population**					
Low Risk		-		-	
High Risk		3.27		0.59–18.64	0.14
**Age**		1.00		0.94–1.07	0.93
**Biological Sex**					
Male		-		-	
Female		1.18		0.21–4.84	0.85

## Discussion

This study underscores the potential role of RIPLs as noninvasive biomarkers of systemic vascular dysfunction, particularly in the context of cardiovascular risk stratification. The findings not only demonstrate that raw RIPL counts differ between high- and low-risk groups, but also that minimal RIPL presence (0–1 RIPLs) is common in the low-risk population and elevated RIPL counts (≥ 2 RIPLs) become significantly more frequent in individuals with high cardiovascular risk, as indicated by CAC scores ≥ 300. This reinforces the notion that RIPLs are a manifestation of chronic systemic vascular pathology and are closely tied to the broader landscape of vascular health, including age, hypertension, and T2DM among other contributors to adverse vascular health.

Furthermore, the univariate analyses demonstrated that elevated RIPL counts are significantly associated with high CAC scores and a greater burden of comorbid diseases. That patients with high CAC scores surprisingly had lower total cholesterol likely was the result of statin use in the high-risk population. Additionally, the observed lack of an independent relationship between RIPLs and CAC scores in multivariate analyses is consistent with the influence of shared comorbidities, such as T2DM, HTN, and smoking, on both coronary and retinal vascular systems. These comorbid conditions are well-documented drivers of systemic vascular dysfunction, and their impact on both large and small vascular beds further highlights the interconnected nature of the cardiovascular system [31–35]. Therefore, it is unsurprising that no independent relationship was identified in this study between RIPLs and CAC scores, as both imaging findings are a downstream result of chronic vascular dysfunction by broader systemic disease, but are not pathophysiologically related. These findings highlight the importance of considering RIPLs as part of a comprehensive assessment of systemic vascular health to assist with characterizing subclinical disease or diagnosing systemic conditions like hypertension [12], carotid artery stenosis [19, 20], or atrial fibrillation [22].

RIPLs represent a unique opportunity to evaluate systemic microvascular health through a noninvasive, accessible, and relatively cost-effective modality — OCT imaging. Unlike invasive imaging techniques, OCT and OCTA imaging allow for the direct visualization of retinal microvascular changes and ischemia, offering a surrogate window into the systemic microvascular environment. Given the retina’s physiologic similarities to other vascular beds, the presence of RIPLs may offer complementary insights into the status of capillary beds, which are unavailable in other forms of CVD risk prediction [32–34]. Notably, we hypothesize that RIPLs can provide novel, complementary data to CAC scores. CAC scores reflect calcified macrovascular atherosclerotic disease, indicating chronic plaque calcification and advanced atherosclerotic burden, whereas RIPLs offer additional insights into microvascular dysfunction and injury, that aren’t assessed in coronary CT scans.

Furthermore, these findings also emphasize the broader systemic implications of chronic ischemic insults. Both elevated CAC scores and RIPL presence are markers of vascular damage, and their coexistence may signify a heightened risk of major adverse cardiovascular events (MACEs), especially given the connection between RIPLs and stroke [16–18] (RIPLs correlate with stroke in individuals with atrial fibrillation as well as with cerebral small vessel disease in individuals with single subcortical infarction) and myocardial infarctions [21]. The degenerative nature of small-scale vascular infarctions underscores the importance of early detection and intervention to prevent further progression and systemic organ dysfunction. The detection of RIPLs in clinical practice should prompt consideration of further evaluation for underlying cardiovascular disease, particularly in patients without a known diagnosis. In such situations, direct communication between ophthalmologists and primary care physicians or cardiovascular specialists might be warranted. Previous studies have shown that patients with unexplained RIPLs who undergo cardiovascular workups are often found to have undiagnosed cardiovascular disease [11]. This suggests that RIPLs could serve as a novel risk enhancer for ASCVD, identifying individuals who might benefit from more aggressive cardiac risk factor management and surveillance. This supports a multidisciplinary approach to managing systemic vascular health, integrating insights from ophthalmology, cardiology, and primary care to address the complex interplay of factors contributing to microvascular and macrovascular disease. It is possible that artificial intelligence tools might be developed to flag patients with RIPLs or even pre-RIPL changes, and such tools might allow for earlier work-up of these patients.

## Limitations

Although this study links increases in number of RIPLs to increases in CVD risk, this study also has several limitations. The cross-sectional design does not allow for the evaluation of the progression of RIPL profiles as cardiovascular risk changes over time. Additionally, intermediate CAC scores (1–299) were excluded, leaving a gap in understanding the relationship between RIPLs and cardiovascular risk in this intermediate-risk population. Another limitation is the potential for selection bias, as patients included in this study likely underwent ocular imaging for clinical reasons, which may have influenced their RIPL profiles. However, this effect would likely be similar across both low- and high-risk groups, with an unknown impact.

## Future directions

Future research should continue to examine the relationship between RIPLs and systemic vascular health. The possibility of a dose-dependent relationship between RIPL counts and CAC scores should be examined, as this might allow for prediction of chronic cardiac dysfunction from retinal microvascular imaging. A prospective evaluation of the progression of RIPLs over time in relation to changing CAC scores and cardiovascular risk would be important in understanding the temporal development of RIPLs as patients’ cardiovascular statuses change over time.

Additionally, studies examining mortality and cardiovascular event rates among high- versus low-RIPL populations in comparison to CAC scores would assess the prognostic value of RIPLs, more directly assessing whether RIPLs offer more information on a patient’s microvascular health and if OCT imaging may serve as a complement to current risk prediction metrics. Finally, future work should assess whether interventions targeting systemic vascular dysfunction, such as antihypertensive or cardiometabolic disease-targeted therapies, reduce the rate of RIPL development and correlate to improved systemic vascular outcomes. Together, these efforts will refine the utility of RIPLs as a noninvasive biomarker of systemic vascular health, inform multidisciplinary strategies for early intervention and risk prevention, and pave the way for RIPLs to be implemented in non-Ophthalmologic clinics.

## Conclusion

This study provides insight into the association between RIPLs and CAC scores. These findings suggest that although RIPLs and CAC frequently occur together in older adults with multiple health conditions, RIPLs do not independently predict CAC scores in this cohort. Further studies must be performed for RIPLs to reach the clinical realm and be used as a complement to current forms of CVD risk estimation. Regardless, this study adds to the growing evidence of RIPLs identified on OCT imaging being a sign of cardiovascular disease. 

## Data Availability

The datasets generated and analyzed in this study are not publicly available due to institutional policies on clinical data and imaging. De-identified data and analysis materials may be available upon reasonable request, subject to Northwestern University IRB review and a data use agreement.

## Abbreviations

AF: Atrial fibrillation
AHA: American Heart Association
AIC: Akaike Information Criterion
ASCVD: Atherosclerotic Cardiovascular Disease
CAC: Coronary artery calcium
CAS: Carotid artery stenosis
CKD: Chronic kidney disease
CT: Computed tomography
CVD: Cardiovascular disease
DCP: Deep capillary plexuses
EMR: Electronic medical records
HTN: Hypertension
ICP: Intermediate capillary plexuses
INL: Inner nuclear layer
MACEs: Major adverse cardiovascular events
OCT: Optical coherence tomography
OCTA: OCT-angiography
ONL: Outer nuclear layer
OPL: Outer plexiform layer
OR: Odds ratio
PAD: Peripheral arterial disease
PAMM: Paracentral acute middle maculopathy
PREVENT: Predicting Risk of CVD Events
RIPLs: Retinal ischemic perivascular lesions
SCP: Superficial capillary plexuses
T2DM: Type 2 diabetes mellitus
